# Impulsivity and self-harm in adolescence: a systematic review

**DOI:** 10.1007/s00787-016-0915-5

**Published:** 2016-11-05

**Authors:** Joanna Lockwood, David Daley, Ellen Townsend, Kapil Sayal

**Affiliations:** 1 Division of Psychiatry & Applied Psychology, Institute of Mental Health, University of Nottingham, Innovation Park, Triumph Road, Nottingham, NG7 2TU UK; 2 Centre for ADHD and Neurodevelopmental Disorders Across the Lifespan, (CANDAL) Institute of Mental Health, University of Nottingham, Nottingham, UK; 3 School of Psychology, University of Nottingham, Nottingham, UK

**Keywords:** Self-harm, Non-suicidal self-injury, Impulsivity, Adolescence, Urgency

## Abstract

Research supports an association between impulsivity and self-harm, yet inconsistencies in methodology across studies have complicated understanding of this relationship. This systematic review examines the association between impulsivity and self-harm in community-based adolescents aged 11–25 years and aims to integrate findings according to differing concepts and methods. Electronic searches of EMBASE, MEDLINE, PsychINFO, CINAHL, PubMed and The Cochrane Library, and manual searches of reference lists of relevant reviews identified 4496 articles published up to July 2015, of which 28 met inclusion criteria. Twenty-four of the studies reported an association between broadly specified impulsivity and self-harm. However, findings varied according to the conception and measurement of impulsivity and the precision with which self-harm behaviours were specified. Specifically, *lifetime* non-suicidal self-injury was most consistently associated with *mood*-*based* impulsivity-related traits. However, *cognitive* facets of impulsivity (relating to difficulties maintaining focus or acting without forethought) differentiated current self-harm from past self-harm. These facets also distinguished those with thoughts of self-harm (ideation) from those who acted on thoughts (enaction). The findings suggested that mood-based impulsivity is related to the initiation of self-harm, while cognitive facets of impulsivity are associated with the maintenance of self-harm. In addition, behavioural impulsivity is most relevant to self-harm under conditions of negative affect. Collectively, the findings indicate that distinct impulsivity facets confer unique risks across the life-course of self-harm. From a clinical perspective, the review suggests that interventions focusing on reducing rash reactivity to emotions or improving self-regulation and decision making may offer most benefit in supporting those who self-harm.

## Introduction

### Self-harm and the extent of the problem in adolescence

Self-harm, defined here as intentional self-injury or self-poisoning irrespective of motivation or intent [1], is a significant problem affecting young people. Though estimates suggest around 25,000 adolescents present to hospital annually with self-harm in England and Wales [2], the often hidden and unreported nature of self-harm suggests that these are conservative estimates [3, 4]. Community-based studies in the UK reveal that around 13–15.5% of adolescents aged 13–18 years report a lifetime incidence of self-harm [5–8]—a high prevalence mirrored in findings from cross-national epidemiological surveys of youth [4, 9, 10]. While rates of self-harm show a consistent peak around 14–17 years [11] and the onset and cessation of self-harm typically occur around this age [4, 12], the behaviour is common across adolescence—here recognised as the broad developmental period spanning 11–25 years [10]. In fact, rates of self-harm in this age group are estimated to be three times those of older adult populations [13]. The high prevalence, coupled with a significant risk of recurrence [14] and strong links to completed suicide [10, 15], underlines the scale of the problem in young people.

Cross-study examinations need to address heterogeneity in the conception and measurement of ‘self-harm’. Well-documented disparities exist between ‘non-suicidal self-injury’ (NSSI) which is seen as distinct from suicidal behaviour and an intent-free conception of ‘self-harm’ that rejects this dichotomy favouring a dimensional perspective on suicidal intent [1, 3, 16]. Intent-free definitions recognise the strong association between self-injury and suicidality, and that the motivations that underlie self-harm behaviour may be multiple, changing, and unclear [1, 16]. Beyond broad conception, studies vary in their definition of what constitutes an NSSI or self-harm act as well as in their application of pre-established criteria [5]. Variable discrimination of self-harm (e.g., recency/frequency of behaviour) makes it further problematic to summarise associations.

### Impulsivity as a risk factor in self-harm

Impulsivity has been identified as a risk factor for self-harm behaviour [17, 18]. A recent review and meta-analysis across clinical and non-clinical mixed age populations [19] found greater levels of self-reported impulsivity in those who engaged in non-suicidal self-injury compared to those who did not. Yet, the relationship between impulsivity and self-harm is not always evidenced, and studies focusing on the association within adolescent populations have revealed inconsistencies in clinical and community populations [5, 20]. The mixed pattern of results is explained in part by variation in the conception and assessment of impulsivity across studies. The same term has been used to depict trait-based personality conceptions of impulsivity (such as the tendency towards rash or unplanned acts, sensation seeking, or difficulty maintaining focus) assessed via self-report scales [e.g., 21–24], alongside state-based conceptions of impulsivity related to behavioural inhibition and typically assessed through lab-based measures, such as the stop-signal task (SST) [25, 26]. That impulsivity is not a unitary construct, but encompasses a number of distinct unidimensional components is well documented [27–29]. Meaningful interpretation of the relationship between impulsivity and an outcome, such as self-harm, relies on adequate specification of which component of impulsivity is under scrutiny, not least because separate impulsivity-related constructs will vary in the magnitude of relationship with outcomes [27, 28, 30, 31] and thus their clinical utility in predicting self-harm. Usefully, the heterogeneity underpinning trait impulsivity has been clarified within one organisational model derived from an exploratory factor analysis of widely used self-report assessment measures. The UPPS-R [27] sets out separate pathways to impulsive behaviour: sensation seeking (SS)—a preference for intense, novel or risky experiences; lack of premeditation (LPM)—acting rashly without due regard to the consequences; lack of perseverance (LPS)—the tendency to abandon goal-directed behaviours; and mood-based dispositions to urgency—rash action in response to negative affect (negative urgency, NUR). More recently, rash action in response to positive affect (positive urgency, PUR) was incorporated (UPPS-P) [32]. The specificity offered by the multidimensional UPPS model allows increased predictive utility when examining impulsivity as a risk factor for self-harm [30].

### Theoretical and wider context of understanding

Impulsivity has been proposed as theoretically important in pathways to self-harm. Theoretical and empirical work suggests that the primary function of self-harm is affect-regulation, i.e., young people self-harm to regulate their emotions, most often to decrease negative emotional states [12, 34–36]. In parallel, Urgency Theory suggests that some individuals, in the presence of heightened negative affect, are more likely to act rashly [33]. The goal of relief from negative affect may drive impulsive behaviour for short-term gain over long-term objectives [37]. Hence, impulsivity (negative urgency) may increase an individual’s vulnerability to engage in a readily accessible though maladaptive behaviour, such as self-harm, to moderate affect [33]. Successful implementation of this strategy in alleviating distress leads to the negative reinforcement of self-harm [35]. Evidence is accumulating of a strong association between negative urgency and NSSI [19]. Other models of self-harm propose a role for impulsivity. The Integrated Motivational–Volitional (IMV) model [38] sets out a tripartite diathesis-stress framework that charts the relationship from background stressors, the development of ideation and intent, and the translation of thoughts into behaviour. The model proposes that impulsivity acts as a proximal volitional moderator to self-harm, bridging the intention-behaviour gap. A distal role for impulsivity in self-harm is also proposed [39, 40] in which individuals with elevated impulsivity may as a consequence experience more painful and provocative experiences over time. Through habituation, they may then have a dampened response to the aversive nature of self-harm which contributes to the maintenance of the behaviour. Summarising the support for these theoretical models within the present review may prove informative. There are important gaps in our understanding of the wider context within which impulsivity relates to self-harm. Results from studies that have taken into account the influence of correlates, such as depression, are inconsistent [6, 41], and limited focus has been given to moderation or mediation designs that may delineate alternative pathways of influence [42]. Furthermore, few studies have examined associations beyond cross-sectional inquiry [43], which makes any causal influence of impulsivity hard to establish.

### Goals of the present review

Review findings [19] across a broad sampling frame suggest that impulsive individuals may be at increased risk of NSSI, but concede that differences in the conception and measurement of these constructs hamper conclusions. The present study aims to extend this understanding with some distinctions in approach. (1) Evidence is examined for an association between impulsivity and *self*-*harm* or *NSSI.* This broad focus is important given that disentangling suicidal intent and self-injury is complicated. (2) Associations are examined in adolescent community-based populations given the high prevalence and onset of self-harm in young people. (3) Particular attention is given to the impact of conception and assessment approach, the precision of measurement, and the comprehensive context of examination.

## Methods

### Identification of relevant studies

A literature search covering articles published up to 6th July 2015 was conducted with the assistance of an information specialist using the following databases: EMBASE, MEDLINE, and PsycINFO via OVID, CINAHL via EBSCOhost, PubMed, and The Cochrane Library via Wiley Online Library. Search keywords, collected through literature review, experts’ opinion, and controlled vocabulary comprised combinations of (1) variants of *impulsivity*, or *impulsiveness* or *impulsive behaviour* and (2) a self-harm term, including variants of *self*-*harm*, or *self*-*injury*, or *deliberate self*-*harm*, or *self*-*destructive behaviour*, or *self*-*mutilation*, or *self*-*poisoning*, or *parasuicide*, or *self*-*inflicted injury*. (The specific search strategy for each database is available from JL.) Studies were also identified through a hand search of the reference lists of included studies and related reviews. Articles were screened by JL initially on the basis of titles and abstracts and then by JL and DD through a full text assessment according to the following set criteria. Peer reviewed studies written in English were included which reported a psychometrically validated measure of impulsivity and indicated self-harm behaviour in community-based samples of 11–25 years. Self-harm behaviours and ideation were included irrespective of suicidal intent. Studies were required to examine the relationship between self-harm and impulsivity. All empirical study designs were included with the exception of case studies and single case designs given their limited generalisability and high potential for bias. Disagreements were resolved by discussion between raters and input from an independent third party was not necessary.

## Results

**Fig. 1:**
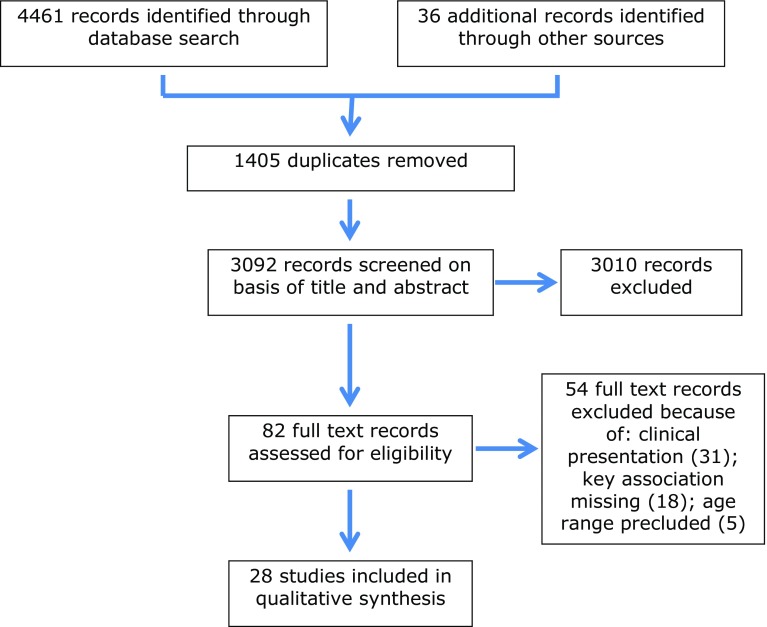
PRISMA diagram showing study selection process

A total of 4496 articles were identified. Exclusion of duplicates and non-relevant abstracts provided 82 full text records assessed for eligibility. Fifty-four records were excluded on the basis of clinical presentation (31 records); missing key association (18 records), or precluded age range (5 records). The remaining 28 studies were subject to descriptive synthesis. Given the variety of study designs and variation in methods of assessment a meta-analysis was not feasible. A PRISMA flowchart recording each stage of the search process is provided in Fig. 1. In a number of cases, the same source studies were included given differentiation in design or outcome measure: in four cases, authors published follow-up studies utilising the same sample [44, 45] and [6, 46], or a subset of an earlier cohort [41, 43] and [47, 48], with the subsequent analysis focused on a different research outcome. Five included studies derived from one international survey data set [4]: four analysed separate country-based subsets [5, 49–51]; the remaining study provided analysis across the complete data set, but pursued a different research question [52]. An additional study [53] drew on the combined data set of two included studies [6, 7] but again focused on a separate research question. Table 1 lists included studies and reports population details, measures, and key results.

**Table 1:** Characteristics of studies included in the systematic review

Study/country	Population		Measurement		Nature of relationship	Quality score
	Source	Gender/age/ethnicity	Impulsivity	NSSI/self-harm		
NSSI studies						
Allen and Hooley [64] USA	Online experiment	*n* = 64 *F* (71%) 69% white (mean age = 23)	SNAP Stop-signal task	Single question. *Lifetime* incidence—intent not established	NSSI group did not report higher levels of trait impulsivity compared to controls, but did demonstrate poorer inhibitory control over *rapid* behavioural response to negative image [*F* (1,62) = 5.17, *p* = .03]; but no broad deficit indicated by SSRT	4 (moderate)
Arens and Gaher [56] USA	Uni/college concurrent	*n* = 407 *F* (65%) 93.6% white (mean age = 20.33)	UPPS-R	DSHI *lifetime* incidence	NSSI positively correlated with NUR [*b* = .048, *p* = .007]; negatively correlated with LPM and LPS. Urgency mediates the relationship between child maltreatment and NSSI presence	6 (good)
Bresin et al. [58] USA	Uni/college EMA diary	*n* = 61 *F* (38) *M* (29) (mean age = 19.58)	UPPS-R Urgency only	DHSI *lifetime* incidence and *daily NSSI urge*	For individuals high in NUR, high daily sadness was a significant predictor of increased NSSI urge (OR 3.93). So was upset, and fear, but not daily negative affect or guilt	6 (good)
Claes and Muehlenkamp [60] BEL	High schools concurrent	*n* = 613 *F* (60.4%) 17.3% NSSI (mean age = 16.38)	UPPS-P	SIQ-TR *lifetime* incidence. Examined NSSI methods	NSSI positively correlated with NUR (rs = .29) and PUR (rs = .18). LPM positively correlated with severe cutting	5 (moderate)
Di Pierro et al. [48] Italy	High school concurrent	*n* = 267 *F* (70.4%) past-year NSSI *n* = 36 (mean age = 17.03)	Youth questionnaire	SIQ-TR *lifetime* incidence and *severity* (high = 5 + cases of *past-year* NSSI)	Impulsivity (lack of premeditation) positively associated with *lifetime* presence of NSSI, but negatively associated with severity of NSSI *p* < .001. Findings held in multivariate analysis	6 (good)
Di Pierro et al. [47]* Italy	High school concurrent	*n* = 30 *F* (63.3%) (mean age = 16.63)	Youth questionnaire	SIQ-TR *lifetime* incidence	Impulsivity (lack of premeditation) moderated the association between negative-high-arousal states before and after NSSI *p* = .03	5 (moderate)
Dir et al. [59] USA	Uni/college concurrent	*n* = 430. NSSI (215) *F* (77.7%) white 74.8% (mean age = 22.36)	UPPS-P	DHSI *lifetime* incidence Also examined frequency, variety and years of NSSI	DSH group was significantly higher than the non-DSH group on LPM (*p* = .01), PUR (*p* < .001) and NUR (*p* < .001). Higher NUR was associated with increased frequency, method variety, years of NSSI controlling for other UPPS dimensions, self-control and affective lability	7 (good)
Fikke et al. [61] Norway	High school experiment	*n* = 97 *F* (75.3%) (mean age = 14.7)	Stop-signal task	FASM. 2 incidents of *past* **-** *year* NSSI. Also *severity* of behaviour	Those with low NSSI had overall higher scores than controls *p* = .02 and those with high NSSI *p* = .01 on SST variables. Students with low NSSI had higher number of SST errors than controls, and higher SSRT scores than those with high NSSI	5 (moderate)
Glenn and Klonsky [41] USA	Uni/college concurrent	*n* *=* 168 *F* (71%) 82 endorsed self-injury (mean age = 19.5)	UPPS (short) Stop-signal task	ISAS *Lifetime incidence* and current (past year) vs historical	Those who self-injured differed from controls on NUR *d* = .53, LPM *d* = .40 and SS *d* = .35, but not LPS. Effect held for NUR and LPM when controlling for depression, anxiety, alcohol abuse. *Current* self-injury associated with higher LPS *d* = .63, but no differences in NUR, LPM or SS. *Frequency* correlated with LPS and LPM. No difference between groups on SST	6 (good)
Glenn and Klonsky [43] USA^a^	Uni/college longitudinal	*n* *=* 81 *F* (51.9%) baseline *n* *=* 51 *F* (72.5%) follow-up (mean age = 19)	UPPS (short)	ISAS *lifetime* incidence, *frequency* and *current* (past 6 month) vs historical	*Lifetime NSSI frequency* was positively associated with LPS at baseline—but not the other UPPS dimensions. LPS was not a significant predictor of NSSI frequency between baseline and follow-up	6 (good)
Liang et al. [57] China	School concurrent	*n* *=* 31 *F* ( 49.1%)NSSI (446), NSSI + SA (48), SA (20) (mean age = 13.92)	BIS-11 (Chinese)	Self-harm questionnaire *lifetime* (NSSI), suicide attempt (SA) and (NSSI + SA)	*Lifetime* self-harm groups (NSSI) and (NSSI + SA) associated with increased impulsivity compared to NOSH *p* < .0083. (NSSI + SA) had higher Impulsivity scores than (NSSI) only	5 (moderate)
Liu and Mustanski [55] USA	Online longitudinal	*n* *=* 246. NSSI = 15.4% *F* (50.8%). LGBT youth (mean age 18.30).	BIS-11; BSSS	ARBA. Single item *recent* (6 month) cutting. Suicidal intent not established	Multivariate HLM of predictors of NSSI revealed greater *past 6* *month* cutting was associated with sensation seeking *p* = .04 but not BIS-11; suicidal ideation was associated with BIS-11 but not sensation seeking	5 (moderate)
Mullins-Sweatt et al. [62] USA	Uni/college concurrent	*n* *=* 211 79% white *F* (68%) NSSI (87) (mean age = 19.83)	UPPS-P	DHSI *lifetime* incidence	NSSI group reported higher NUR (*d* = .58), LPM (*d* = .47) and LPS scores (*d* = .48) than non-NSSI group	4 (moderate)
Ogle and Clements [13]	Uni/college concurrent	*n* *=* 500 NSSI = 44 *F* (100%) white (88%) (mean age = 18.83)	UPPS-R	DSHI *lifetime* incidence	In univariate tests those engaging in NSSI were significantly higher in NUR (*m* *=* 29.18) than controls (*m* *=* 26.08); no difference in the other facets of impulsivity	4 (moderate)
Peterson et al. [42] USA	Uni/college concurrent	*n* *=* 884 *F* (82%) NSSI = 154 (mean age = 19.16)	UPPS-R	DHSI short-form *lifetime* incidence. Also frequency and current v historical	NUR predicted NSSI engagement (OR 1.047). A three-way interaction was found between NUR, DT and depression with high NUR + depression predicting higher levels of NSSI in those with low DT. A main effect of LPM was found but no interaction effect	7 (good)
Peterson and Fischer [44] USA	Uni/college longitudinal	*n* *=* 489 *F* (100%) white (75.8%) NSSI 23.8% 8 mo *n* *=* 209 (mean age = 18.62)	UPPS-R	DHSI short-form *lifetime* incidence	At baseline direct paths from NUR to NSSI were significant *p* *<* .01. No other impulsivity facet predicted NSSI. NUR did not contribute incremental variance to increases in symptoms over time beyond baseline NSSI or UPPS scales	4 (moderate)
Taylor et al. [45] USA^a^	Uni/college concurrent	*n* *=* 429 *F* (77.2%) white (79%) lifetime *n* *=* 120 current = 33 (mean age = 19.77	UPPS-R	DSHI short-form *lifetime* also current (past month) vs lifetime and motives	Main effect for NUR (*p* *<* .001), LPM (*p* *<* .001)and LPS (*p* *<* .016) on *lifetime* presence of NSSI, but not SS. No association between impulsivity and motives for SI or current NSSI status	4 (moderate)
Rodav et al. [63] Israel	School concurrent	*n* *=* 275 *F* (49.%9) past-year NSSI *n* *=* 57 (mean age = 14.81)	BIS-11 (Hebrew)	OSI-F—*past-year* incidence	Those reporting NSSI had higher impulsivity compared to controls (*p* *<* .0001) but impulsivity was no longer significant when controlling for age, depression and suicidal ideation	7 (good)
Self-harm studies						
De Leo and Helier [50] Australia	School concurrent	*n* *=* 3757. *n* *=* 233 (mean age = 15.4)	Plutchik short form (6 items)	CASE LCQ *past year* and *lifetime* incidence	Impulsivity was associated with past-year self-harm in univariate analysis. No association found in multivariate analysis. Time to engage measures showed that more than a third engaged in SH less than an hour after first thinking about it	7 (good)
Hawton et al. [5] UK	School concurrent	*n* *=* 6020. Past year SH = 398. *F* (75.1%) (age mainly 15–16)	Plutchik short form (6 items)	CASE LCQ *past-year* and *lifetime* incidence	Impulsivity was associated with *past-year* self-harm in boys (OR 1.22) and girls (OR 1.20). In multivariate analysis impulsivity distinguished females (OR 1.10) but not males	7 (good)
Madge et al. [52] Europe and AUS	School concurrent	*n* *=* 30,477 (mainly 15–16)	Plutchik short form (6 items)	CASE LCQ *past year* and *lifetime* incidence also ideation/single/multiple episodes	Increased severity of self-harm history associated with greater impulsivity. Impulsivity was independently associated with self-harm thoughts only (OR 1.06), single episode (OR 1.10), multiple episode (OR 1.13). Only impulsivity among psychological correlates distinguished ideation from single episode self-harm	7 (good)
McMahon et al. [49] Ireland	School concurrent	*n* *=* 3881 *F* (52%) (mainly 15–16 years)	Plutchik short form (6 items)	CASE LCQ. *Past year* and *lifetime* incidence	*Past-year* self-harm associated with impulsivity in boys (OR 1.30) and girls (OR 1.19) in univariate analysis, but only retained independent significance in multivariate analysis for boys (OR 1.17)	7 (good)
O’Connor et al. [7] NI	School concurrent	*n* *=* 3596 white (98.1%) *F* (47.8%) SH *n* *=* 394 *F* (261) (mean age = 15)	Plutchik short form (6 items)	Modified version of CASE LCQ *lifetime SH*	In univariate analysis increased impulsivity was associated with *lifetime* SH in girls (OR 1.20) and in boys (OR 1.25). In multivariate analysis impulsivity was independently associated with lifetime SH in boys only (OR 1.18)	7 (good)
O’Connor et al. [6] SCO	School concurrent	*n* *=* 2008 *F* (53%). SH *n* *=* 272 *F* (209) (mean age = 15.4)	Plutchik short form (6 items)	Modified version of CASE LCQ *lifetime SH*	*Lifetime* SH was associated with increased impulsivity in boys (OR 1.18) and girls (OR 1.17) in univariate analysis but no longer an independent predictor in multivariate analysis	7 (good)
O’Connor et al. [53] Subsample SCO + N.I^a^	School concurrent	*n* *=* 5604 *n* *=* 628 enactors; 675 ideators. *F* (49%) (mainly 15–16 years)	Plutchik short form (2 items)	Modified version of CASE LCQ *Lifetime SH* and *ideation*	*Lifetime* self-harm ideators and enactors differed from controls (OR 1.17 and 1.29) and each other (i.e. enactors were more impulsive (OR 1.13) in univariate analysis. This effect did not retain significance in multivariate multinomial logistic regression	6 (good)
O’Connor et al. [46] Subsample SCO study^a^	School longitudinal	*n* *=* 737 *F* (367) *n* *=* 500 at 6 month FU (mean age = 15.2)	Plutchik short form (6 items)	Modified version of CASE LCQ *lifetime* SH and status at 6 mo follow-up	In univariate analysis impulsivity at baseline was not associated with first time self-harm between T1 and T2 or with repeat self-harm between T1 and T2. But those who failed to complete measures at T2 were significantly more impulsive than completers	7 (good)
Portzky et al. [51] Holland/Belgium	School concurrent	*n* *=* 4431 *F* (49.5%) SH = 243 (mean age 15.45)	Plutchik short form (6 items)	CASE LCQ *past year* and lifetime incidence	In univariate analysis impulsivity was significantly associated with *lifetime* self-harm, but did not retain significance in multivariate analysis	7 (good)
Rawlings et al. [65] UK	Uni/college concurrent	*n* *=* 1350 *F* (71.7%) SH = 85 (mean age 23.62)	BIS-11 and UPPS-P	Two questions *recent* (4 weeks)	No relationship between (BIS-11, LPM and LPS) and self-harm. Affective impulsivity (NUR/PUR) higher in those reporting self-harm (OR 2.0). Relationship between affective impulsivity and SH mediated by depression/anxiety	7 (good)

*SNAP* schedule for nonadaptive and adaptive personality—2 [22], *SIQ-TR* self-Injury questionnaire treatment related [79], *ISAS* inventory of statements about self-injury [12], *FASM* functional assessment of self-mutilation [36], *ARBA* aids-risk-related behaviour among adolescents [80], *OSI-F* Ottawa self-injury inventory functions [81], *DSHI* deliberate self-harm inventory [82], *BSSS* brief sensation seeking scale [24], *LCQ* lifestyle and coping questionnaire, UPPS facets: *NUR* negative urgency, *PUR* positive urgency, *LPM* lack of premeditation, *LPS* lack of perseverance, *SS* sensation seeking, *SST* stop-signal tasks

* Indicates repeat sample or subsample

### Indicators of study quality

A quality rating system was developed in line with criteria recommended for non-clinical study assessment [54]. A four point quality scale included: (1) representativeness of a general population (0–2 points); (2) use of standardised measures of impulsivity (0–2 points); (3) robust criteria specified for indicators of self-harm (0–2 points); and (4) attempts to deal with confounds (0–2 points). Ratings ranged from good (6–8 points), moderate (3–5 points) to low (0–3 points) depending on level of criteria met or the robustness of the study’s conclusions. The average quality score was 5.9 (mean) or 7 (mode) with 19 studies receiving a good quality rating (Table 1). Study quality was independently assessed by JL and DD with 100% agreement.

### Study characteristics

Excluding repeated data sets, a total of 47,055 young people were sampled of which 4114 (8.7%) endorsed self-harm. Of those, 3021 (73.4%) were school based with mean ages ranging from 13.9 to 17.0 years; 1023 (24.7%) were university based with mean ages ranging from 18.8 to 23.6 years; and 70 (1.69%) were derived from online community samples with mean ages ranging from 14.4 to 23.0 years.

### General finding and study synthesis

In line with recent findings [19], an association between broadly specified impulsivity and a self-harm outcome of interest was found in 24/28 studies. All exceptions were longitudinal examinations [43, 44, 46, 55]. Following methodological and conceptual scrutiny, three overarching themes were identified and endorsed through consensus agreement: (1) *conception and assessment approach*; (2) *precision of measurement*; and (3) *influence of more comprehensive study design.*

### Conception and assessment approach—self-harm

The majority of studies (18 out of 28 or 64%) conceptualised self-harm as *non*-*suicidal self*-*injury* (*NSSI*). All but two [55, 56] conferred status via self-report in which the absence of suicidal intent was clearly specified to respondents [13, 41–45, 47, 48, 56–63]. Assessment of NSSI behaviour was broadly comparable across these 18 studies (see Table 1 for instrument details). 15 out of the 16 studies (excepting Liang et al. [57]) that specified the absence of suicidal intent used commonly employed instruments in which the psychometric properties have been validated in adolescent samples and which detailed a range of behaviours against which respondents could identify their own self-injurious behaviour. Listed behaviours across these 16 studies consistently endorsed cutting, burning, and hitting behaviours. One study [61] provided a further categorisation of severity of injury (as degree of tissue damage). Two studies established self-injury on the basis of single questions that did not specify the absence of intent [55, 64]. When analysing the relationship between impulsivity and NSSI, nine studies classified self-injury as the presence or absence of one or more *lifetime* NSSI behaviours [13, 44, 48, 56, 57, 59, 60, 62, 64]; four cases compared *lifetime* to a more recent indication of self-injury [41–43, 45]. Four studies required at least one [47, 63] or two [61] incidents of self-injury in the *past year*, or one or more incident of cutting in the past 6 months [55]. Five out of 18 NSSI studies (27%) analysed self-injury severity or frequency [41, 43, 47, 59, 61]. One study [58] analysed a daily indication of urge to self-injure.

The remaining ten studies adopted an intent-free definition of ‘self-harm’ and highly comparable approaches to assessment. Nine studies employed the lifestyle and coping questionnaire (LCQ) developed in clinical and community adolescent populations for the Child and Adolescent Self-Harm in Europe (CASE) [4] study. This provided a consistent definition of self-harm as a deliberate act of self-injury or self-poisoning irrespective of motivation or suicidal intent [5–7, 46, 49–53]. Five CASE studies [5, 49–52] adhered to a rigorous methodology in which those identified as self-harming on the basis of one or more self-reported incidents of *past*-*year* self-harm were asked to provide a description of their most recent act for classification as self-harm by three independent raters against a standardised criteria. Participants failing to provide this description were excluded from subsequent analysis. An additional grouping of studies followed a modified version of this methodology in which rated descriptions were not required for inclusion [6, 7, 46, 53]. These studies examined the association between impulsivity and *lifetime* self-harm, excepting a 6-month prospective study [46]. In two out of nine *self*-*harm* studies (22%), an examination of ideation or repetition was included (52, 53]. One study employed a single item question to ascertain the presence of the *past-month* self-harm [65].

### Conception and assessment approach—impulsivity

The UPPS scale was the most commonly endorsed assessment tool measuring trait impulsivity in 12 examinations. Six cases adopted the 45-item UPPS-R [13, 42, 44, 45, 56, 62]; three utilised the 59-item UPPS-P [59, 60, 65]. Both scales have good reliability and validity [27, 32]. In two cases [41, 43], the 16-item short form (UPPS-R) was employed which has demonstrated comparable psychometric properties to the long form [66]. One study [58] focused on a single UPPS-R subscale. All nine concurrent full-scale UPPS examinations found a significant association between at least one impulsivity subscale and a self-harm outcome, and these were maintained in all multivariate examinations (*n* = 6) underlining a broad instrument-level consistent utility in this assessment tool. Urgency subscales were the most consistently-associated impulsivity facets associated with the presence of *lifetime* self-injury in full-scale UPPS-R and UPPS-P examinations, and signalled exclusively in four cases [13, 44, 60, 65]. In all cases except one [65], studies adopting UPPS scales defined self-harm as non-suicidal self-injury. Less consistent results were found from studies utilising trait-based instruments that reflect non-mood-based cognitive conceptions of impulsivity as a predominantly rash action with little planning or forethought. Of the four studies utilising the Barratt Impulsivity Scale (BIS-11) [22], two found no relationship between impulsivity and either NSSI [55] or self-harm [65]; two studies demonstrated associations with NSSI [57, 63], but in one case, this association no longer held when controlling for age, depression, and suicidal ideation [63]. Null findings resulted from the single examination using the SNAP (Schedule for non-Affective and Affective Personality) impulsivity scale [22, 64]. Utilising the Youth Questionnaire [67], Di Pierro et al. [47] demonstrated a significant association between *lifetime* NSSI and impulsivity that held in the context of other psychological correlates (anxiety and depression), although the non-validated Italian version and poor internal validity are noted. A mixed pattern of findings resulted from examinations (*n* = 9) using the Plutchik impulsivity scale [23]. Across the full international CASE study sample (*n* = 30,477), an overall significant but small (partial *η* ^2^ = <.01) univariate association between impulsivity and *past*-*year* self-harm was evidenced [52]. However, examining findings by CASE country, although univariate associations between impulsivity and self-harm were demonstrated [5, 49–51], these associations were attenuated in multivariate analysis to non-significance [50, 51] or retained significance for only a subset of girls [5] or conversely boys [49]. Similarly, O’Connor et al. found that significant associations between *lifetime* self-harm and impulsivity were negated completely [6, 53] or retained only for boys [7], and were not demonstrated in longitudinal examinations [46]. Only one UPPS-based study [41] found evidence of an association between sensation seeking (SS) and self-injury, not held in multivariate analysis. Sensation seeking measured by the brief sensation seeking scale [25] was associated with an increased risk of cutting in an LGBT (Lesbian, Gay, Bisexual, Transgender) sample [55].

A state-based conception of impulsivity (as response inhibition) was examined by three studies [41, 61, 64]—in each case assessed by the stop-signal task [25]. For Glenn and Klonsky [41], the SST did not distinguish students with *lifetime* self-injury from controls, they were, however, distinguished by UPPS measures of impulsivity. Fikke et al. [61] similarly found that compared to controls, impaired inhibitory control was not evident in students, whose self-injury endorsed ‘high severity’ behaviours (characterised by severe cutting and burning). However, those endorsing ‘low severity’ behaviours (such as biting and bruising) did make more inhibitory control errors. In both cases, these studies examined behavioural impulsivity using neutral stimuli. When the SST was manipulated to include stimuli to specifically evoke unpleasant emotional reactions, Allen and Hooley [64] demonstrated that compared to controls individuals who self-injured exhibited poorer inhibitory control over negative images, but did not differ in response to neutral stimuli. Notably, this study did not find that non-affective trait impulsivity (SNAP) was associated with self-injury. Furthermore, where stimuli specifically related to NSSI (images of cutting), those who self-injured demonstrated *enhanced* inhibitory control compared to controls, responding similarly when presented with positively valenced images and cutting images.

### Precision of measurement

The second focus of synthesis examines the extent to which the relationship between impulsivity and self-harm varies according to how precisely constructs are measured. Two studies [41, 45] examined if UPPS facets relate differentially to current versus past NSSI. Glenn and Klonsky [41] found that negative urgency and to a lesser extent lack of premeditation, but *not* lack of perseverance, differentiated undergraduates with a *lifetime* history of self-injury from those without. Conversely, only LPS, and not NUR or LPM, differentiated those with *current* (past year) versus *historical* NSSI. Taylor et al. [45] similarly found that undergraduates who self-injured differed from controls on NUR, LPM and, to a lesser extent, LPS, but conversely demonstrated no difference in these variables amongst those endorsing *current* versus *historical* self-injury, albeit with a more stringent definition of the current self-injury (past month). Nonetheless, Taylor et al. conclude that impulsivity may be implicated in the initiation but not the maintenance of self-injurious behaviours. In longitudinal examinations, Peterson and Fischer [44] demonstrated that though associated at baseline, NUR provided no incremental validity over and above the initial expression of self-injury at an 8-month follow-up. Nor were UPPS-based impulsivity facets found by Glenn and Klonsky to predict the course of self-injury over 1 year [43]. Only two further studies examined the influence of impulsivity facets on presentations of self-harm over time [46, 55]. In neither case was an association found between cognitive impulsivity and self-harm at baseline or 6-month follow-up, although indicated cases were small (*n* = 18) [46]; samples were focused on a specialist and potentially non-generalisable group [55], and the internal reliability of the impulsivity measure was poor [46]. Interestingly, O’Connor et al. [46] reported that those who failed to complete measures at follow-up had significantly higher levels of cognitive impulsivity (but not other psychological variables) than those retained in follow-up analysis, which may have influenced the null findings. For the present purposes, trait impulsivity reveals limited prospective utility.

Two studies demonstrated that non-affect-based impulsivity (Plutchik) discriminated between self-harm ideation and enaction. Madge and colleagues [52] revealed that impulsivity (but no other psychological correlate) differentiated between self-harm thoughts and past-year single episodes (i.e., those thinking about self-harm reported significantly lower impulsivity than those acting on their thoughts), suggesting an explanatory role for impulsivity in the initiation of self-harm acts. Similarly, O’Connor et al. [53] demonstrated that, relative to ideators, self-harm enactors reported significantly higher impulsivity, albeit with a small effect size and reliance on just two scale items. These findings were not retained in multivariate analysis. A number of studies considered how impulsivity interacts with more precise assessments of the frequency and severity of self-harm [41, 48, 52, 61]. A differential mechanism of influence for cognitive versus affective facets of impulsivity was demonstrated by the finding that UPPS-based LPS and LPM (but not NUR) predicted the ‘frequency’ of self-injury among undergraduates, i.e., an inability to think through consequences of behaviours or remain focused was positively related to the total number of behaviours endorsed [41]. Relatedly, Madge et al. [52] demonstrated a dose–response effect, with increased severity of the past-year self-harm (no behaviour, ideation, single episode, and multiple episodes) associated with increased cognitive impulsivity. However, Dir et al. [59] found that only the NUR subscale (and not facets relating to cognitive deficits) related to frequency of self-harm; while Di Pierro et al. [48] found that though positively associated with *lifetime* presence of self-injury, lack of premeditation was *negatively* associated with a past-year summation of behaviour, i.e., less frequent self-injurers were more likely to be impulsive.

### Influence of a more comprehensive study design

The final focus considers the impact of more complex study design and analytic approaches on key findings. A mixed pattern of results was found for studies in which covariates were considered in the analysis. The impulsivity-self-harm relationship was retained in the presence of depression or anxiety [41, 43, 48, 52]; affective lability and self-control [59]; gender, negative affect, and child maltreatment [56]; and self-esteem [52]. Elsewhere, the inclusion of covariates appeared to dampen or negate any independent association between impulsivity and self-harm. Notably, studies adopting the lifestyle and coping questionnaire, which included a range of social, psychological and stressful life event factors, resulted in attenuation in strengths of association in multivariate analysis for partial subsets [5–7, 49] or complete samples [50, 51]. In non-CASE studies, adjustment for depression and anxiety [65] and age, depression, and suicidal ideation [63] also negated findings. Rawlings and colleagues [65] further demonstrated that depression and anxiety mediated the relationship between UPPS dimensions (NUR and PUR) and self-harm. Other mediation analysis found that NUR (but not other UPPS facets) mediated the relationship between child maltreatment and NSSI [56]. In analysis of the moderating influence of distinct impulsivity constructs on self-harm outcomes, Di Pierro et al. [47] demonstrated that the successful regulation of affect following NSSI alters as a function of lack of premeditation in a sample of students endorsing past-year self-injury. They found that when negative-high-arousal affect states (nervous, anxious, and angry) pre-NSSI increased, those with higher levels of impulsivity had a greater increase in negative-high-arousal affect and less of an improvement in positive affect state (relief) post-NSSI, relative to those low in impulsivity, i.e., those less able to evaluate the consequences of their actions had greater difficulty regulating their negative affect. Impulsivity then may be implicated, where affect-regulation is not successful. By comparison, Bresin et al. [58] used a daily diary study design to examine the influence of impulsivity (high or low NUR) on general and specific facets of negative affect in predicting the *urge* to self-injure. They revealed that for individuals high in NUR, daily sadness (but not general negative affect, or guilt) was a significant predictor of urge to self-injure, but for those low in NUR, there was no relationship between sadness and NSSI urge. While urge to self-injure is not necessarily a precursor to NSSI engagement, nonetheless, these studies [47, 58] provide useful explication of the context in which a theorised outcome (affect-regulation following NSSI) might hold. In a comprehensive modelling of covariates, Peterson et al. [42] examined the extent to which impulsivity (NUR or LPM) interacts with distress tolerance (the cognitive appraisal of the one’s ability to cope with distress) and depression, to predict lifetime NSSI. They revealed that undergraduates high in NUR and depression, but with low distress tolerance, were more likely to report lifetime NSSI; however, no significant interaction was found for LPM. Thus, a propensity to act rashly in the presence of negative affect, combined with low perceived ability to cope with that affect, may increase NSSI vulnerability.

## Discussion

Findings from this review suggest that, broadly defined, impulsivity relates to self-harm behaviour in community-based populations of adolescents aged 11–25, with evidence of an association with a self-harm outcome of interest in 24 out of 28 studies. However, this relationship varies in accordance with how precisely constructs are measured and the wider context of measurement and analysis. Considering the consistency and reliability of the constructs used raises some interesting points. Studies were conceptually divided in defining self-harm, with 57% overtly precluding suicidal intent, although the majority of cases examined a range of similar indicated behaviours through common validated instruments. Importantly, conceptual distinctions did not explain the heterogeneity in review findings, as associations with impulsivity were demonstrated across *NSSI* and *self*-*harm* studies. Impulsivity facets were more consistently associated with self-injury classified as non-suicidal overall, but a relationship between impulsivity and suicidality was revealed in NSSI studies and underscores the difficulties inherent in separating self-harm and suicidality. Namely, students endorsing both NSSI and suicide attempt were found to have significantly higher trait impulsivity than those endorsing NSSI only [57]; impulsivity was found to relate to suicidal ideation but not NSSI [55]; and the association of impulsivity to NSSI was found to disappear when controlling in part for suicidal ideation [63]. These findings signal that impulsivity is important to understanding both self-injury and suicidality in young people and that this relationship may vary across levels of suicidal thinking and behaviour.

Although the *NSSI* studies by definition endorsed a narrower conception of self-injury, the *self*-*harm* studies largely employed more stringent inclusion criteria. The requirement to fulfil the robust CASE study methodology brings transparency, consistency, and comparability across a large subset of studies in this review. The additional CASE stipulation to corroborate behaviour is conceptually advantageous, although as noted elsewhere, it risks an underestimate of behaviours, where the provision of a description may be considered too personal and unwelcome by respondents [6, 7]. It is problematic that, for the most part, reviewed studies examined solely a *lifetime* (*n* = 11) or *past-year* (*n* = 8) indication of self-harm, which provides a very broad-brush indication of behaviour, (and which crucially fails to delineate that distress underpinning behaviours may have abated). Arguably, this approach may be obscuring associations between impulsivity and self-harm. The six studies that did examine distinctions in self-harm/NSSI (frequency/number of methods/symptoms) found associations with cognitive, affective, and behavioural impulsivity [41, 43, 48, 52, 59, 61]. Combining methodological stringency with a finer grained examination of self-harm may reveal a clearer pattern of association.

In terms of *impulsivity*, most cases in the present review adopted a trait-based conception of an underlying personality disposition that can be captured via questionnaire. This provides conceptual comparability but reflects an over-reliance on single respondent self-report, which brings an associated bias. Demonstrably, the choice of assessment tool is important in explicating the relationship between trait impulsivity and self-harm. The most commonly endorsed and consistently supported tool was the multidimensional and well-validated UPPS model which found either a direct association [13, 41–45, 59, 60, 62, 64, 65] or indirect association [58] in 12 examinations. All except one [65] of the UPPS examinations were based within NSSI studies. By contrast, in all but one *self*-*harm* study [52] impulsivity as assessed by the Plutchik scale did not retain an independent association for complete samples [6, 50, 51] or subsets of boys [5, 7] or girls [49]. The likelihood that it is the greater utility of the UPPS multidimensional instrument that drives the more consistently found association between *NSSI* (rather than *self*-*harm*) and impulsivity in the present review, is reinforced by Rawlings and colleagues [65] who were able to demonstrate that though UPPS subscales predicted *self*-*harm* behaviour in a sample of undergraduates, BIS-11 subscales did not. A key conceptual distinction between the UPPS and BIS-11 measures lies in the former’s inclusion of affect-based facets of impulsivity compared to the cognitive facets reflected by the latter (relating to lack of planning and perseverance, or remaining focused on tasks). Thus, mood-based facets may in part underlie the strong association between UPPS-assessed impulsivity and NSSI. In support, urgency subscales were the UPPS facets most consistently associated with NSSI/self-harm. Yet, a key driver in the inconsistent overall pattern of trait *cognitive* impulsivity may be psychometrically based. Although the full Plutchik Impulsivity Scale has demonstrated good psychometric properties in adolescent samples [68], the present studies drew on a short form of six [5–7, 46, 49–51] or just two [53] items. The psychometric properties of other tools (such as the BIS-11 and Youth Questionnaire) have not been extensively examined in community samples [47, 69].

In fact, where examinations extend beyond *lifetime* or *past-year* indicators of self-harm, a more finely grained picture of the association between cognitive impulsivity and self-harm emerges. Facets examining lack of planning and forethought were implicated in the frequency of self-injury [41, 48, 52] and in differentiating self-harm status, such as ideation from enaction [52, 53] or current from historical NSSI [41]. Thus, the review supports a dual pathway of risk for the influence of trait impulsivity on self-harm, through rash reactivity to (predominantly negative) affect and deficits in cognitive control. The question of when these respective components of impulsivity exert their influence over the life-course of self-harm, however, remains. Of interest, Glenn and Klonsky [41] produced evidence of a differential role for mood-based and non-mood-based facets, with NUR and LPM implicated in the lifetime presence but not continued maintenance of self-injury—a pattern reversed for LPS. Their interpretation that NUR may lead to the adoption of self-injury, but that LPS is associated with an inability to resist the urge to self-injure once behaviour has been initiated, is persuasive (for recent empirical support, see [70]). That impulsivity may be more implicated in the initiation than the maintenance of self-harm is here supported cross-sectionally and longitudinally [43, 45]. Further longitudinal studies, which consider the interaction between impulsivity facets, are needed to clarify if the risk for maintained self-harm is reduced for those individuals high in trait urgency, but low in traits related to cognitive deficits. Risk models which account for the transaction between trait-based impulsivity and broader cognitive processing (e.g., [71]) may further clarify onset and maintenance risk for self-harm. For example, while it is theorised that those high in NUR may recruit a maladaptive behaviour in the service of immediate short-term relief from negative affect [33], this process may be mediated at initiation by the ‘expectancy’ that affect can be regulated and will deliver relief (see [28, 37]). The understanding that relief will not last may mean that long-term maintenance of self-harm relates less to the urge to regulate affect and more to deficits in self-control, decision making, and the momentary ability to enlist an alternative coping response (see [71]). The present evidence that cognitive appraisal of distress interacted with urgency to predict NSSI [42], or NUR is negatively associated with self-control [59], underlines the utility of examining trait-based risk within a wider cognitive context.

Inconsistent findings resulted from the three state-based examinations of inhibitory control and self-harm, with support for behavioural (but not trait) impulsivity [64]; partial support for behavioural impulsivity [61]; or no support for behavioural impulsivity (but support for trait impulsivity) [41]. The negligible association between trait and behavioural measures of impulsivity is well documented [30] and, arguably, pathways to self-harm from individuals that display elevated levels of trait impulsivity, or those endorsing situationally impulsive acts, may have little correspondence. It is possible of course that behavioural measures are providing an accurate assessment of impulsivity, and, as noted previously [19], the greater association between trait impulsivity and self-harm reflects the bias of an underlying confound, such as ‘perceived’ impulsivity, perhaps self-validated by the inclusion of an impulsivity item in a self-harm questionnaire [20, 58]. The present findings in fact suggest that behavioural impulsivity is important under conditions of negative affect. Though employing a-contextual measures of emotional responding as a proxy for self-harm, Allen and Hooley’s [64] manipulation of the SST to include non-neutral stimuli, nevertheless, offers a conceptually stringent test of association and provides objective support for the relevance of emotional reactivity in the relationship between state impulsivity and self-harm. In this light, it is interesting to conjecture methodologically on the endorsement of the UPPS scale. Importantly, the UPPS scale measures the traits that lead to impulsive behaviour, and hence, reactivity is specified within the context of an emotional state: “*When* I am upset I often act without thinking”. As such, the model’s predictive utility may derive in part from its ability to account for intra-individual variation in behaviour in relation to situational (state based) factors to a greater degree than traditional trait-based scales. Problematically, a temporal disconnect remains across many designs (i.e., measuring a baseline assessment of impulsivity with a past indication of self-harm) that cannot control for such variation. Comprehensive designs that account for the momentary context within which predispositions to impulsivity play out, and move beyond binary state vs. trait distinctions (see [58]) may offer greater utility in delineating pathways to self-harm. Relatedly, in a recent study using sequence analysis techniques to examine factors leading to self-harm, impulsivity (identified by the item “I did it on impulse without planning’ and which may relate to trait disposition or momentary state) was identified as the only proximal factor immediately preceding the first ever and most recent episode [73]. Support was also found in the present review for the influence of trait impulsivity in the translation of self-harm thoughts into behaviour [52, 53] as theorised by the Integrated Motivational–Volitional model of self-harm/suicidal behaviour [38]. Importantly, discriminating between intention and enaction has been identified as a critical area for self-harm research [72]. That trait impulsivity is closely associated with behavioural enaction speaks to the possible transaction between trait and state conceptions of impulsivity. In light of the present findings, tests of the IMV model with the multidimensional UPPS tool, and which extend to longitudinal examination, are an obvious next step to further clarify the role of both affect and non-affect-based facets of impulsivity in the initiation and longer term maintenance of self-harm.

In support of the interrelation between urgency theory and the affect-regulation function of self-harm [12, 33, 34], findings suggest that the relationship between self-harm outcomes and impulsivity is best understood in terms of how impulsivity relates to mood and the short-term management of emotion. Complex models of analysis comprehensively specified this emotional context for impulsive reactivity and revealed: a moderating influence of lack of premeditation on the successful regulation of high-arousal affect following NSSI [47]; that sadness relates to NSSI urge for those high in negative urgency [58]; that vulnerability to NSSI is most significant for those who not only tend to react rashly to negative mood, but also perceive themselves as unable to cope with negative mood [42]; and that child maltreatment may result in a tendency to deal with negative affect impulsively [56]. These studies pinpoint the role of cognitive and affective processing in links between trait impulsivity and self-harm. Importantly, findings suggest a differential relationship between facets of impulsivity and separate indices of affect [47, 58] in the affect-regulation process. Given the prominence of the affect-regulation function of self-harm, future research should now build upon these lines of enquiry. It is interesting to reflect on the finding of Allen and Hooley [64] in this context who revealed impaired behavioural inhibition over negatively valenced stimuli, but note that this pattern was reversed when stimuli directly referenced NSSI. The authors suggest that this finding indicates a level of habituation to NSSI at which impulsive reactivity is no longer implicated. Such habituation may in part underlie findings that those endorsing more severe or frequent NSSI demonstrate lower impulsivity than those endorsing ‘less severe’/frequent NSSI [47, 61]. These findings support theories of habituation and that a dampening response to the aversive nature of self-harm may be important to the continuation of self-harm behaviour [39, 40].

The findings from this review have practical implications for clinical treatment. Targeting rash reactivity to intense emotions may be useful in identifying those at increased risk for self-harm. Psychological interventions that teach the regulation or tolerance of emotion and focus on rational decision making over emotional response may be usefully directed at those initiating behaviour or indicating ideation. Distress tolerance and problem-solving skills are core components of dialectical behaviour therapy (DBT) [74] and the application of a modified version of DBT for adolescents (DBT-A) [75] has shown promise in trials with reductions in self-harm frequency sustained at 1-year follow-up [76, 77]. Treatment retention and engagement with follow-up are a recognised problem among those who self-harm [78] and may be a particular challenge for those high in impulsivity who experience difficulties remaining on task or focusing on long-term goals. Keeping patients in treatment is an explicit goal of DBT and evidence of good treatment retention for DBT-A is noteworthy [76]. Nonetheless, DBT-A requires adolescent and family adherence over a 16-week duration. Promisingly, a brief 40-min intervention (Therapeutic Assessment) based on cognitive analytic therapy approaches and delivered at initial hospital presentation has demonstrated long-lasting improvements in adolescent engagement with treatment [78] and may promote increased motivation for adherence with interventions offered. Reference to the UPPS multidimensional tool may help clinicians clarify the nature of risk for individuals [31]. For those who tend to act with little forethought, or have difficulty remaining on task, cognitive-regulation techniques which focus on the outcomes of rash action and highlight long-term goals may be helpful, particularly in treating maintained self-harm/NSSI. Less is currently known of positive urgency as a risk for self-harm, though a focus on the consequences of behaviour may help to mitigate risk relating to positive arousal [31, 33].

There are limitations to the conclusions that can be drawn from the review. An over-reliance on cross-sectional designs across mainly *lifetime* indications of self-harm limits understanding of the long-term relationship, or direction of effects, between self-harm and impulsivity. Future studies should account for the wider and temporal context of how affect and cognitive control of emotion may alter behavioural response across the life course of self-harm. Greater focus on the differential impact of positive versus negative urgency facets is also warranted. In addition, research should examine the self-harm-impulsivity relationship across gender and culture given the differential gender effects outlined [5, 7, 49], and the lack of cultural diversity reflected across studies.

The present review builds on earlier work [19] in demonstrating an association between impulsivity facets and NSSI/self-harm, here specifically in community-based adolescents. Methodologically, findings indicate the necessity of clearly defined constructs, specified precisely, to clarify understanding of this relationship. More broadly, examination of the interplay between different facets of impulsivity and a nuanced account of self-harm that considers intention, enaction, frequency and severity would clarify the strength of this relationship. Research beyond associative studies is needed to explain when and why the relationship between impulsivity facets and self-harm is expressed and how it relates to regulatory functioning.

## References

[1] Kapur N, Cooper J, O’Connor RC, Hawton K (2013). Non-suicidal self-injury v. attempted suicide: new diagnosis or false dichotomy?. Br J Psychiatry.

[2] Hawton K, Fagg J, Simkin S, Bale E, Bond A (2000). Deliberate self-harm in adolescents in Oxford, 1985–1995. J Adolesc.

[3] Hawton K, Bergen H, Kapur N, Cooper J, Steeg S, Ness J (2012). Repetition of self-harm and suicide following self-harm in children and adolescents: findings from the multicentre study of self-harm in England. J Child Psychol Psyc.

[4] Madge N, Hewitt A, Hawton K, Wilde EJ, Corcoran P, Fekete S (2008). Deliberate self-harm within an international community sample of young people: comparative findings from the Child and Adolescent Self-harm in Europe (CASE) Study. J Child Psychol Psyc.

[5] Hawton K, Rodham K, Evans E, Weatherall R (2002). Deliberate self harm in adolescents: self report survey in schools in England. BMJ.

[6] O’Connor RC, Rasmussen S, Miles J, Hawton K (2008). Self-harm in adolescents: self-report survey in schools in Scotland. Br J Psychiatry.

[7] O’Connor RC, Rasmussen S, Hawton K (2014). Adolescent self-harm: a school-based study in Northern Ireland. J Affect Disord.

[8] Morey Y, Mellon D, Dailami N, Verne J, Tapp A (2016). Adolescent self-harm in the community: an update on prevalence using self-report survey of adolescents aged 13–18 in England. J Public Health.

[9] Giletta M, Scholte R, Rutger CME, Ciarano S, Prinstein MJ (2012). Adolescent NSSI: a cross national study of community samples from Italy, the Netherlands, and the United States. Psychiatr Res.

[10] Muehlenkamp J, Havertape L, Plener P (2012). International prevalence of adolescent non-suicidal self-injury and deliberate self-harm. Child Adolesc Psychiatry Ment Health.

[11] Whitlock J (2012). Self-injurious behavior in adolescents. Plos Med.

[12] Klonsky ED, Glenn CR (2009). Assessing the functions of non-suicidal self-injury: psychometric properties of the inventory of statements about self-injury (ISAS). J Psychopathol Behav.

[13] Ogle RL, Clements CM (2008). Deliberate self-harm and alcohol involvement in college-aged females: a controlled comparison in a nonclinical sample. Am J Orthopsychiatr.

[14] Hawton K (2007). Self-harm in England: a tale of three cities. Multicentre study of self-harm. Soc Psychiatry Psychiatric Epidemiol.

[15] Owens P (2002). Fatal and non fatal repetition of self harm: a systematic review. Br J Psychiatry.

[16] Andover M, Morris B, Wren A, Bruzzese M (2012). The co-occurrence of NSSI and attempted suicide among adolescents: distinguishing risk factors and psychosocial correlates. Child Adolesc Psychiatry Ment Health.

[17] Herpertz S, Steinmeyer SM, Marx D, Oidtmann A (1995). The significance of aggression and impulsivity for self-mutilative behavior. Pharmacopsychiatry.

[18] Simeon D, Stanley B, Frances A, Mann JJ, Winchel R, Stanley M (1992). Self-mutilation in personality disorders: psychological and biological correlates. Am J Psychiatry.

[19] Hamza CA, Willoughby T, Heffer T (2015). Impulsivity and nonsuicidal self-injury: a review and meta-analysis. Clin Psychol Rev.

[20] Janis IB, Nock MK (2009). Are self-injurers impulsive?: results from two behavioral laboratory studies. Psychiatr Res.

[21] Barratt E (1959). Anxiety and impulsiveness related to psychomotor efficiency. Percept Mot Skills.

[22] Clark LA, Simms LJ, Wu KD, Casillas A (2008). Manual for the schedule for nonadaptive and adaptive personality (SNAP-2).

[23] Plutchik R, Van Praag H (1989). The measurement of suicidality, aggressivity and impulsivity. Prog Neuro-Psychopharmacol Biol Psychiatry.

[24] Hoyle RH, Stephenson MT, Palmgreen P, Lorch EP, Donohew RL (2002). Reliability and validity of a brief measure of sensation seeking. Personal Ind Differ.

[25] Logan GD, Cowan WB (1984). On the ability to inhibit thought and action: a theory of an act of control. Psychol Rev.

[26] Dick DM, Smith G, Olausson P, Mitchell SH, Leeman RF (2010). Review: understanding the construct of impulsivity and its relationship to alcohol use disorders. Addict Biol.

[27] Whiteside SP, Lynam DR (2001). The five factor model and impulsivity: using a structural model of personality to understand impulsivity. Pers Individ Dif.

[28] Smith GT, Cyders MA (2016). Integrating affect and impulsivity: the role of positive and negative urgency in substance use risk. Drug Alcohol Depend.

[29] Cyders MA, Coskunpinar A (2012). The relationship between self-report and lab task conceptualizations of impulsivity. J Res Pers.

[30] Lynam DR, Miller JD, Bornovalova MA, Lejuez CW (2011). Testing the relations between impulsivity-related traits, suicidality, and nonsuicidal self-injury: a test of the incremental validity of the UPPS model. Personal Disord.

[31] Zapolski TCB, Settles RF, Cyders M, Smith GT (2010). Borderline personality disorder bulimia nervosa, antisocial personality disorder, ADHD, substance use: common threads, common treatment needs, and the nature of impulsivity. Indep Pract.

[32] Cyders MA, Smith GT, Spillane NS, Fischer S (2007). Integration of impulsivity and positive mood to predict risky behavior: development and validation of a measure of positive urgency. Psychol Asses.

[33] Cyders MA, Smith GT (2008). Emotion-based dispositions to rash action: positive and negative urgency. Psychol Bull.

[34] Chapman AL, Gratz KL, Brown MZ (2006). Solving the puzzle of deliberate self-harm: the experiential avoidance model. Behav Res Ther.

[35] Klonsky ED (2007). The functions of deliberate self-injury: a review of the evidence. Clin Psychol Rev.

[36] Nock MK, Prinstein MJ (2004). A functional approach to the assessment of self-mutilative behavior. J Consult Clin Psychol.

[37] Tice DM, Bratslavsky E, Baumeister RF (2001). Emotional distress regulation takes precedence over impulse control: if you feel bad, do it!. J Person Soc Psych.

[38] O’Connor RC, O’Connor RC, Platt S, Gordon J (2011). Towards an integrated motivational-volitional model of suicidal behavior. Int handbook of suicide prevention.

[39] Joiner T (2005). Why people die by suicide.

[40] Anestis MD, Soberay KA, Gutierrez PM, Hernández TD, Joiner TE (2014). Reconsidering the link between impulsivity and suicidal behavior. Personal Soc Psychol Rev.

[41] Glenn CR, Klonsky ED (2010). A multimethod analysis of impulsivity in non-suicidal self-injury. Personal Disord.

[42] Peterson CM, Davis-Becker K, Fischer S (2014). Interactive role of depression, distress tolerance and negative urgency on non-suicidal self-injury. Personal Ment Health.

[43] Glenn CR, Klonsky ED (2011). Prospective prediction of nonsuicidal self-injury: a 1-year longitudinal study in young adults. Behav Ther.

[44] Peterson CM, Fischer S (2012). A prospective study of the influence of the UPPS model of impulsivity on the co-occurrence of bulimic symptoms and non-suicidal self-injury. Eat Behav.

[45] Taylor J, Peterson CM, Fischer S (2012). Motivations for self-injury, affect, and impulsivity: a comparison of individuals with current self-injury to individuals with a history of self-injury. Suicide Life Threat Behav.

[46] O’Connor RC, Rasmussen S, Hawton K (2009). Predicting deliberate self-harm in adolescents: a 6-month prospective study. Suicide Life Threat Behav.

[47] Di Pierro R, Sarno I, Gallucci M, Madeddu F (2014). NSSI as an affect-regulation strategy and the moderating role of impulsivity. Child Adolesc Ment Health.

[48] Di Pierro R, Sarno I, Perego S, Gallucci M, Madeddu F (2012). Adolescent nonsuicidal self-injury: the effects of personality traits, family relationships and maltreatment on the presence and severity of behaviours. Eur Child Adolesc Psychiatry.

[49] McMahon EM, Reulback U, Corcoran P, Keeley H, Perry I, Arensman E (2010). Factors associated with deliberate self-harm among Irish adolescents. Psychol Med.

[50] De Leo D, Heller TS (2004). Who are the kids who self-harm? An Australian self-report school survey. Med J Aust.

[51] Portzky G, De Wilde EJ, van Heeringen K (2008). Deliberate self-harm in young people: differences in prevalence and risk factors between The Netherlands and Belgium. Eur Child Adolesc Psychiatry.

[52] Madge N, Hawton K, McMahon E, Corcoran P, De Leo D, de Wilde K (2011). Psychological characteristics, stressful life events and deliberate self-harm: findings from the child and adolescent self-harm in Europe (CASE) study. Eur Child Adolesc Psychiatry.

[53] O’Connor RC, Rasmussen S, Hawton K (2012). Distinguishing adolescents who think about self-harm from those who engage in self-harm. Br J Psychiatry.

[54] Sanderson S, Tatt I, Higgins JPT (2007). Tools for assessing quality and susceptibility to bias in observational studies in epidemiology: a systematic review and annotated bibliography. Int J Epidemiol.

[55] Liu RT, Mustanski B (2012). Suicidal ideation and self-harm in lesbian, gay, bisexual, and transgender youth. Am J Prev Med.

[56] Arens AM, Gaher RM, Simons JS (2012). Child maltreatment and self-harm among college students: testing mediation and moderation models for impulsivity. Am J Orthopsychiatry.

[57] Liang S, Yan J, Zhang T, Zhu C, Situ M, Du N (2014). Differences between non-suicidal self injury and suicide attempt in Chinese adolescents. Asian J Psychiatry.

[58] Bresin K, Carter DL, Gordon KH (2013). The relationship between trait impulsivity, negative affective states, and urge for nonsuicidal self-injury: a daily diary study. Psychiatry Res.

[59] Dir AL, Karyadi K, Cyders MA (2013). The uniqueness of negative urgency as a common risk factor for self-harm behaviors, alcohol consumption, and eating problems. Addict Behav.

[60] Claes L, Muehlenkamp J (2013) The relationship between the UPPS-P impulsivity dimensions and non-suicidal self-injury characteristics in male and female high-school students. Psychiatry J ID 654847 10.1155/2013/654847 PMC3820090 24236284

[61] Fikke LT, Melinder A, Landrø NI (2011). Executive functions are impaired in adolescents engaging in non-suicidal self-injury. Psychol Med.

[62] Mullins-Sweatt SN, Lengel GJ, Grant DM (2013). Non-suicidal self-injury: the contribution of general personality functioning. Personal Ment Health.

[63] Rodav O, Levy S, Hamdan S (2014). Clinical characteristics and functions of non-suicide self-injury in youth. Eur Psychiatry.

[64] Allen KJ, Hooley JM (2015). Inhibitory control in people who self-injure: evidence for impairment and enhancement. Psychiatry Res.

[65] Rawlings J, Shevlin M, Corcoran R, Morriss R, Taylor PJ (2015). Out of the blue: untangling the association between impulsivity and planning in self-harm. J Affect Disord.

[66] Cyders MA, Littlefield AK, Coffey S, Karyadi KA (2014). Examination of a short English version of the UPPS-P impulsive behavior scale. Addict Behav.

[67] Brook JS, Brook DV, Gordon S, Whiteman M, Cohen P (1990). The psychosocial etiology of adolescent drug use: a family interactional approach. Genet Soc Gen Psychol Monogr.

[68] Grosz DE, Lipschitz DS, Eldar S (1994). Correlates of violence risk in hospitalized adolescents. Compr Psychiatry.

[69] Reise SP, Moore TM, Sabb FW, Brown AK, London ED (2013). The Barratt impulsiveness scale-11: reassessment of its structure in a community sample. Psychol Asses.

[70] Riley E, Combs J, Jordan C, Smith GT (2015). Negative urgency and lack of perseverance: identification of differential pathways of onset and maintenance risk in the longitudinal prediction of NSSI. Behav Ther.

[71] Pearson C, Wonderlich S, Smith GT (2015). A risk and maintenance model for Bulimia Nervosa: from impulsive action to compulsive behavior. Psychol Rev.

[72] Klonsky ED, Victor SE, Saffer BY (2014). Nonsuicidal self-injury: what we know, and what we need to know. Can J Psychiatry.

[73] Townsend E, Wadman R, Sayal K, Armstrong M (2016). Uncovering key patterns in self-harm in adolescents: sequence analysis using the card sort task for self-harm (CaTS). J Affect Disord.

[74] Linehan M (1993). Cognitive behavioral treatment of borderline personality disorder.

[75] Miller AL, Rathus JH, Linehan M (2007). Dialectical behavior therapy with suicidal adolescents.

[76] Mehlum L, Tormoen AJ, Ramberg M (2014). Dialectical behavior therapy for adolescents with repeated suicidal and self-harming behavior: a randomized trial. J Am Acad Child Adolesc Psychiatry.

[77] Mehlum L, Ramberg M, Tormoen AJ (2016). Dialectical behavior therapy compared with enhanced usual care for adolescents with repeated suicidal and self-harming behavior: outcomes over a 1-year follow up. J Am Acad Child Adolesc Psychiatry.

[78] Ougrin D, Boege I, Stahl D (2013). Randomised controlled trial of therapeutic assessment versus usual assessment in adolescents with self-harm: 2-year follow-up. Arch Dis Child.

[79] Claes L, Vandereycken W, Goldfarb PM (2007). The self-injury questionnaire-treatment related (SIQ-TR): construction, reliability, and validity in a sample of female eating disorder patients. Psychological tests and testing research trends.

[80] Donenberg GR, Emerson E, Bryant FB, Wilson H, Weber-Shifrin E (2001). Understanding AIDS-risk behavior among adolescents in psychiatric care: links to psychopathology and peer relationships. J Am Acad Child Adolesc Psychiatry.

[81] Nixon M, Cloutier P, Aggrawal S (2002). Affect regulation and addictive aspects of repetitive self-injury in hospitalized adolescents. J Am Acad Child Adolesc Psychiatry.

[82] Gratz KL (2001). Measurement of deliberate self-harm: preliminary data on the deliberate self-harm inventory. J Pscyhopathol Behav Assess.

